# Vegetable Fillers and Rapeseed Oil-Based Polyol as Natural Raw Materials for the Production of Rigid Polyurethane Foams

**DOI:** 10.3390/ma14071772

**Published:** 2021-04-03

**Authors:** Milena Leszczyńska, Elżbieta Malewska, Joanna Ryszkowska, Maria Kurańska, Michał Gloc, Michał K. Leszczyński, Aleksander Prociak

**Affiliations:** 1 Faculty of Materials Science and Engineering, Warsaw University of Technology, Wołoska 141, 02-507 Warsaw, Poland; joanna.ryszkowska@pw.edu.pl (J.R.); michal.gloc.wim@pw.edu.pl (M.G.); 2 Faculty of Chemical Engineering and Technology, Cracow University of Technology, Warszawska 24, 31-155 Cracow, Poland; elzbieta.malewska@pk.edu.pl (E.M.); maria.kuranska@pk.edu.pl (M.K.); aleksander.prociak@pk.edu.pl (A.P.); 3 Faculty of Chemistry, Warsaw University of Technology, Noakowskiego 3, 00-664 Warsaw, Poland; mleszczynski@ch.pw.edu.pl; 4 Institute of Physical Chemistry, Polish Academy of Sciences, Kasprzaka 44/52, 01-224 Warsaw, Poland

**Keywords:** polyurethane foams, renewable sources, rapeseed oil-based polyol, natural oil, vegetable fillers, biocomposites

## Abstract

The reported study concerns the introduction of renewable raw materials into the formulation of rigid polyurethane foams in the quest for the sustainable development of polymer composites. In this study, rigid polyurethane foam composites were prepared using 75 wt.% of rapeseed oil-based polyol and 15 parts per hundred parts of polyol (php) of natural fillers such as chokeberry pomace, raspberry seeds, as well as hazelnut and walnut shells. The influence of the used raw materials on the foaming process, structure, and properties of foams was investigated using a FOAMAT analyzer and a wide selection of characterization techniques. The introduction of renewable raw materials limited reactivity of the system, which reduced maximum temperature of the foaming process. Moreover, foams prepared using renewable raw materials were characterized by a more regular cell structure, a higher share of closed cells, lower apparent density, lower compressive strength and glass transition temperature, low friability (<2%), low water absorption (<1%), high dimensional stability (<±0.5%) and increased thermal stability. The proper selection and preparation of the renewable raw materials and the rational development of the polyurethane recipe composition allow for the preparation of environmentally-friendly foam products with beneficial application properties considering the demands of the circular economy in the synthesis of rigid foams.

## 1. Introduction

The desirable concepts of sustainable development involve the major economic and environmental actions related to the transformation of postproduction waste into raw materials and reduction of the irreversible effects of fossil fuels extraction, which can be facilitated by increasing the content of renewable raw materials in the polymer production [1,2]. The global polyurethane (PU) market based mainly on petrochemical raw materials was estimated to be worth $95.13 billion in 2019 and is forecasted to increase at an annual rate of 12% up to $149.91 billion by 2023 [3]. The PU foams market dominates the PU market with a share of 67% [4]. The major reasons for the high demand for rigid polyurethane foams (RPUFs) are their high mechanical strength with low apparent density, high dimensional stability in a broad temperature range, excellent thermal insulation and sound-absorbing properties, physiological inertness, resistance to the development of fungi and mold, good adhesive properties, resistance to water and weather conditions, and availability in various customized shapes, which allows them to be used in industries such as construction, automotive, furniture, aviation, and cosmetics [3,5].

One of the methods complying with the economic and legal requirements related to environmental protection is limiting the use of petrochemical raw materials in the synthesis of polyurethane foams by using biopolyols based on different vegetable oils such as rapeseed [6,7], tung [8], soybean [9], palm [2], as well as biopolyols made from waste cooking oils [10,11], etc. [12]. These oils have versatile compositions and structures, are affordable, biodegradable, non-toxic, and environmentally friendly, and are soluble in most of industrial solvents [12]. However, literature reports indicate difficulties in the synthesis of PU foams using natural polyols due to their high viscosity, secondary functional groups (of relatively low reactivity), and low hydroxyl numbers. These properties can result in a longer curing time when natural polyols react with isocyanates to produce PU. Moreover, in comparison to PU foams based on petrochemical substrates, they might have inferior properties such as heterogeneous cell structure, difficulty in apparent density control, poor dimensional stability, and low strength, low glass transition temperature, susceptibility to permanent deformation. The mentioned changes may negate the potential benefits related to the use of these cheaper renewable polyols [12,13,14]. Therefore, researchers are making considerable efforts to develop efficient biopolyol synthesis methods and foam formulas in order to produce biofoams with properties comparable or better to those of petroleum-based analogs [15]. For example, Ji et al. reported on the preparation of foams using 25 wt.% of soy-based polyol synthesized from epoxidized soybean oil by oxirane ring opening, which resulted in improvement of mechanical and thermal properties except for dimensional stability [9]. However, upon the increase of the soy-based polyol content to 50 wt.%, the properties of the foams deteriorated due to the low crosslink density due to high steric hindrance related to the presence of benzene rings. In another literature report, thermal insulating RPUFs were prepared using high-functional rapeseed oil-based polyols (fn = 3.6–5.8) [15]. The prepared foams had high closed cell content (>95%), apparent density of ca. 40 kg/m^3^, and thermal conductivity of 21.5–23.3 mW/mK.

Another method allowing for PU foams’ production in accordance with the circular economy idea is the use of plant waste from the agri-food industry for the production of polymeric materials [16]. In the United States, each year, nearly 45 million tons of fresh vegetables, fruits, milk, and grain products are disposed of as waste. According to the Environmental Protection Agency (EPA), the annual cost of recycling such a huge amount of waste is over $1 billion. In Great Britain, the food production waste is over 20 million tons yearly, and each ton of food waste generates 4.5 tons of CO_2_ emissions [17]. According to the data contained in report [17], the share of unused raw materials in the production of fruit and vegetable juices is 30–50%, in the production of vegetable oils—40–70%, and in the case of sugar production from sugar beets, it even reaches 86%.

The abovementioned waste streams are typically utilized in a range of ways such as transformation into fodders and fertilizers, use in the production of distillates, dyes, pectin, as well as incineration for energy recovery [18]. As demonstrated in the recent literature reports, the waste can be a valuable raw material for the production of PU foams [18,19,20,21]. Moreover, the introduction of algal cellulose into PU composites (0.5–1.6 (w/v)) might lead to enhanced properties due to the integration of the natural product into the foam structure, which results from an increased shock absorption capacity of the materials [22]. The influence of the potato protein (PP) on the properties of RPUFs was also investigated, at it yielded foams with enhanced mechanical properties, higher apparent density, comparable thermal conductivity, and lower water uptake compared to a neat foam (0.1 wt.% of PP) [23]. However, the authors indicated that the use of 1–5 wt.% of PP led to the deterioration of the properties of foams, which resulted in a lower number of closed cells and enhanced porosity, lower apparent density, lower compression strength, and higher thermal conductivity.

The properties of the composites are the result of many factors such as size, structure, chemical composition, and adhesion of the additive added to the matrix material. Therefore, the quality of the used additive has a significant impact on the properties of composites with plant-based fillers [21,24,25,26,27]. The structure of fillers differs in the degree of porosity, the arrangement of layers, shape, size and arrangement of individual units. The chemical composition of fillers includes mainly cellulose, hemicellulose, lignin, fats, proteins, tannins, dyes, mineral salts, and water [18,26]. A characteristic feature of lignin is its hydrophobic nature, limiting the ability of plant fibers to bind water and swell. This substance also increases the resistance of fibers to microorganisms. Cellulose, composed of linear D-glucose chains linked with β1-4 glycosidic bonds, is non-toxic, hydrophilic, and biodegradable; it is the main structural component of cell walls [22]. 

Water naturally present in plant-based filler particles is also a chemical blowing agent in the synthesis of PU foams. The amount of foaming agents used in the polyurethane foam preparation determines the apparent density of the final product and is closely related to the quality of the cellular structure of the PU foam and its mechanical properties, dimensional stability, and thermal insulation. Therefore, the water released from natural filler particles should be included in the designed foam formulation in order to maintain high control over the resulting foam properties [28,29].

Another important issue is related to the high prices of natural fillers pretreated using advanced and energy-consuming physical and chemical modification processes, which lead to a significant reduction of profit resulting from the use of waste fillers. Moreover, treatment of raw materials with chemicals that require further utilization negates the environmentally friendly character of the initiative [26]. Therefore, it is advantageous to use fillers of natural origin subjected to the least possible processing steps and operations.

In the current study, rigid polyurethane foam composites were produced using renewable raw materials: rapeseed oil-based polyol and chokeberry pomace, raspberry seeds, as well as hazelnut and walnut shells obtained as waste streams from the agri-food industry. The preliminary treatment of the fillers was limited to the necessary grinding and initial drying without using any additional chemical reagents. The study aimed to determine the effect of the type of plant-based filler and plant oil-based polyol on the course of the PU composition foaming process and the properties of the obtained RPUF composites. An additional aim of the conducted study was the development of foam composites with high renewable raw materials content which exhibit favorable physical, mechanical, and thermal properties. Formulations of biocomposites of PU foams were developed taking into account the water content released from plant additives in order to eliminate any unfavorable effects resulting from a too high proportion of the blowing agent in the composition. Detailed characterization of natural raw materials was performed in order to analyze their chemical composition as well as grain size and morphology. The influence of raw materials on the course of the foaming process, structure and properties of foams was also determined.

## 2. Materials and Methods

### 2.1. Raw Materials for Polyurethane Foams Preparation

Rapeseed oil-based polyol (ROP) with the hydroxyl value of 248 mg KOH/g, acid value of 3.2 mg KOH/g, number average molar mass of 1374 g/mol, water content of 0.16 wt.%, and viscosity of 7512 mPa·s was prepared from a rapeseed oil substrate at Cracow University of Technology using a two-step procedure. Initially, the double bonds in rapeseed oil were epoxidized, followed by the oxirane ring opening by reaction with diethylene glycol (DEG) [30].

Natural fillers—chokeberry pomace (CH), raspberry seeds (R), hazelnut shells (HS), walnut shells (WS) (waste products from the food and agriculture industry)—were supplied by industrial partners involved in the processing of nuts and juices. The natural fillers were mechanically ground using a Retsch ZM 200 centrifugal mill (chokeberry pomace), a MKM6003 mill (Bosch) (raspberry seeds), MUKF-10 mill (Młynpol) (hazelnut shells, walnut shells). The fillers were milled until 100% of the observed grain particle fractions were smaller than 500 µm and then dried at 70 °C to a constant mass using a temperature chamber to remove any water absorbed on the filler surface. Due to the possible presence of the water retained inside the filler particles, which is difficult to remove in low-temperature processes, the weight loss of the predried fillers was measured using a Radwag moisture analyzer. In order to evaluate the possibility of release of the retained water during the high-temperature foaming process, the 2.0 g filler samples were analyzed in the temperature range from 25 °C to 140 °C [18,21]. The results of the analysis indicate the following weight loss of the fillers in the analyzed temperature range: CH = 1.92%, R = 1.78%, HS = 2.01%, WS = 2.03% (measurement time < 120 s).

Polios^®^ 420 PTA-aromatic polyester polyol (hydroxyl value, 433 mg KOH/g, number average molar mass, 330 g/mol, water content, 0.10 wt.%) was supplied by commercial vendor Purinova, Poland. Rokopol^®^ G500, a glycerol-based polyoxyalkylene triol (hydroxyl value, 300 mg KOH/g, number average molar mass, 560 g/mol, water content, 0.10 wt.%), was supplied by PCC Rokita, Poland. Ongronat^®^ TR 4040, a mixture of oligomeric methylene diphenyl diisocyanate (MDI) and MDI isomers containing 32.6 wt.% of free isocyanate groups, was supplied by BorsodChem, Hungary. JEFFCAT^®^ ZF-10 and JEFFCAT^®^ DPA purchased from Huntsman Corporation, The Woodlands, TX, USA were used as catalysts. A silicone surfactant TEGOSTAB^®^ B4900 purchased from Evonik Industries, Germany, was used as a foam structure stabilizer. Distilled water was used as a chemical porophore due to the generation of gaseous CO_2_ upon reaction with isocyanate groups.

### 2.2. Synthesis of Rigid Polyurethane Foams

The synthesis of RPUFs was performed using a single-step method by mixing (1600 RPM, 10 s) the polyol premix containing polyols, catalysts, the surfactant, and the blowing agent (PU_REF; PU_ROP) with the isocyanate component. In the case of biocomposite synthesis, the polyol premix was supplemented with 15 php (parts per hundred parts of polyol) of ground chokeberry pomace (PU_ROP_CH), raspberry seeds (PU_ROP_R), walnut shells (PU_ROP_WS) or hazelnut shells (PU_ROP_HS) (Table 1). The selection of the optimal rapeseed oil-based polyol content was preceded by the synthesis of a series of foams using 0–100 wt.% ROP content (Supplementary Data, Section S1, Table S1). As a result, the foam containing 75 wt.% ROP was selected as a reference formulation for further introduction of plant-based fillers due to its favorable properties such as structure (Figure S1), closed cell content (Figure S2) thermal properties (Figures S3 and S4; Tables S2 and S3), apparent density, friability, water absorption and the dimensional stability (Table S4). The filler content of 15 php was selected based on the results obtained in the previous publications of the authors [21,31,32]. Due to the anticipated release of water from natural filler particles, in line with previous investigations in this area [18,21], the amount of distilled water added to the polyurethane foam composition was adjusted accordingly to the moisture analysis results (Section 2.1) in order to avoid the excess of the blowing agent. Moreover, the water contained in the used polyols was also accounted for in the formula calculations. After mixing the components, the mixture was poured into an open mold. Next, the foams were annealed for 30 min at 70 °C and conditioned at room temperature and 50% relative humidity for 24 h before being removed from the mold. After two weeks, the resulting foams were cut and tested. The resulting RPUFs exhibited apparent density in the range of 62–84 kg/m^3^ and differed in color with regard to the type of natural filler used (PU_REF–yellow, PU_ROP–yellow, PU_ROP_CH–purple, PU_ROP_R/WS/HS–light brown).

### 2.3. Characterization of Raw Materials and Rigid Polyurethane Foams

Ground natural fillers were subjected to the sieve analysis according to Polish Standard PN-EN 933-10:2002.

The content of lignin, hemicellulose, and cellulose was determined in accordance with the PN-92/P-50092 standard as well as literature reports [33]. The content of crude fat was determined by the Soxhlet extraction with chloroform [34].

The dynamic viscosity of the mixtures of polyols and polyols with natural fillers was studied at 25 °C using the shear rates of 0–50 s^−1^ using a Brookfield viscometer model DV-II + Pro equipped with a measuring spindle type SC4-29.

The shape and size of filler particles and foam cells were analyzed using a Hitachi TM3000 SEM (Hitachi Group, Tokyo, Japan) using the accelerating voltage of 5 keV. All of the samples were covered with a layer of palladium and gold using a Polaron SC7640 sputter coater (Quorum Technologies Ltd, Laughton, UK) for 100 s at 10 mA before SEM imaging.

Foam analysis system FOAMAT (Format Messtechnik GmbH, Karlsruhe, Germany) was used for the study of the foaming process, allowing for the investigation of key parameters such as reaction temperature and dielectric polarization during the foaming process of PU systems [35].

The distributions of cell and filler particle sizes as well as overall porosity were studied using an Xradia 400CT tomograph (2 × 2 × 2 cm cubic samples, (Zeiss, Jena, Germany) equipped with computer software suited for 3D image reconstruction. The X-ray beam was generated using 40 kV accelerating voltage and 10 W power. During the analysis, 1261 frames were collected, which represented 180° rotation of the sample. Frame were collected every 3 s. The #LE1 filter was used and the detector location was selected in order to enhance the phase contrast of the collected images. Total scanning time for each sample was about 4 h, including reference frames of the air every 8 min. After completion of data collection, the 3D images were reconstructed using the X-Radia software (Zeiss, Jena, Germany)), introducing the center shift and beam hardening parameters if needed. The estimation of total porosity as well as of cell and filler particle size distributions was conducted by analysis of the resulting 3D models (estimated image resolution around 20 µm) using CT software, utilizing the binarization threshold and defect removal functions.

The Fourier transform infrared spectroscopy (FTIR) study was conducted using a Nicolet 6700 spectrometer (Thermo Electrone Corporation, Waltham, MA, USA). Spectral data were collected as a sum of 64 scans in the range of 4000–400 cm^−1^. The CO_2_ and H_2_O impact on the data was removed with the baseline correction. The results were analyzed using the OMNIC8.2.0 software by ThermoFisher Scientific Inc.

Thermogravimetric analysis (TGA) was performed with TGA Q500 (TA Instruments, New Castle, PA, USA) using 10 ± 0.5 mg of samples which were heated in a nitrogen atmosphere from room temperature up to 800 °C at the rate of 10 °C/min. Data analysis was performed using the Universal Analysis 2000 software, version 4.7 A, by TA Instruments.

The differential scanning calorimetry (DSC) was conducted using DSC Q1000 (TA Instruments, New Castle, PA, USA) using samples (5 ± 0.2 mg) placed in hermetic aluminum cups. The experiments consisted of heating the samples at 10 °C/min, cooling at 5 °C/min, and then reheating at 10 °C/min. The Universal Analysis 2000 software, version 4.7 A, by TA Instruments was used to determine glass transition temperatures.

The friability, apparent density, compressive strength, and closed cell content of the RPUFs were determined according to standards ASTM C-421, ISO 845, ISO 844, and ISO 4590, respectively.

The water absorption analysis as well as the dimensional stability test were conducted at 40 °C. For each material, three samples (50 × 50 × 25 mm^3^) were weighed and measured, followed by immersion in water for 24 h. After taking the samples out of the water and removal of water from the external sample surface, the samples were weighed and measured again. This allowed for the calculation of both the dimensional stability (the ratio of the difference in size before and after the procedure to the initial size) as well as water absorption (ratio of the absorbed water to the initial volume of the sample). The test was carried out in accordance with the procedure developed by Fampur Adam Przekurat Company (Bydgoszcz, Poland).

## 3. Results

### 3.1. Analysis of the Natural Raw Materials and the Processing Parameters of the Used Compositions

The properties of foam materials are sensitive to changes in composition and preparation parameters, including compatibility and viscosity of the materials. The quality of filler dispersion, size, shape, and the possibility of anchoring in the polymer matrix have influence on the cell structure and properties of foams. The introduction of solid particles into the polyol premix might significantly alter bubble nucleation conditions, which directly affects the size, shape, and number of pores, as well as pore wall thickness in the final materials [25,36]. Therefore, the detailed characterization of the used fillers is crucial for rational foam material design.

#### 3.1.1. Sieve Analysis of the Natural Fillers

**Figure 1:**
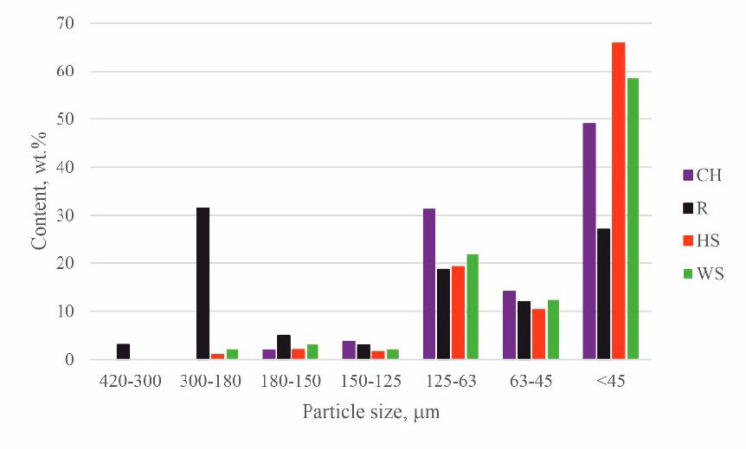
The grain size analysis results.

The results of the sieve analysis show that hazelnut shells were characterized by the highest share of fraction <45 µm in size (Figure 1). Walnut shells exhibited similar fraction distribution to HS, but the share of particles >45 µm in size was slightly higher compared to HS. The size of the ground chokeberry pomace did not exceed 180 µm and the highest percentage (~50%) of particles were smaller than 45 µm. Raspberry seeds were characterized by the widest distribution of the filler size, including ~32% within the fraction size of 180–300 µm. The reason for the observed differences in the filler particle size distribution is related to the use of different grinding mills and the susceptibility of the fillers to fragmentation due to their different chemical composition, morphology, and physical properties.

#### 3.1.2. Chemical Composition of the Natural Fillers

In the study, the share of cellulose, hemicellulose, lignin, and raw fat in CH and R was determined (Table 2). The chemical composition of WS and HS fillers was determined in the previous report [21,34].

The results of the analysis show that the highest share of crude fat is present in raspberry seeds, while the lowest is observed in hazelnut shells. Moreover, the data indicate that the chokeberry pomace features the highest share of hydrophobic lignin and a lower total proportion of hydrophilic components (cellulose, hemicellulose) compared to the other analyzed fillers, in which the ratio of hydrophilic to hydrophobic components is close to ~3:2.

#### 3.1.3. Thermal Analysis of the Natural Fillers

**Figure 2:**
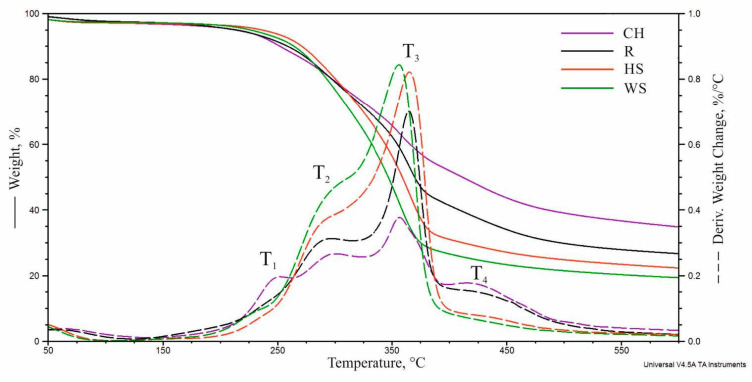
TG and DTG curves of the natural fillers.

Figure 2 presents the TG (weight change) and DTG (derivative weight change) curves obtained as a result of thermal decomposition of the fillers. After the release of the water contained in the fillers, the proportion of which was determined using a moisture analyzer (Section 2.1), the decomposition of individual components was observed: lignin (160–500 °C), hemicellulose (220–315 °C), and cellulose (315–400 °C) [21,37,38]. The DTG curves obtained for WS and HS show a similar degradation pathway with a clearly visible signal resulting from cellulose degradation with a T_3_ maximum and an inflection point with no additional maximum indicating the degradation of hemicellulose (T_2_). The DTG curve of R is characterized by a clearly visible signal from hemicellulose (T_2_) due to the highest share of hemicellulose in the filler among the analyzed additives and an additional signal (T_4_) without a clear maximum in the range of 400–500 °C resulting from lignin decomposition. The degradation of the chokeberry pomace occurs in four stages, with a T_1_–T_4_ maxima, which may result from the highest content of lignin in the filler among the analyzed additives. The residue after combustion at the temperature of 600 °C for the analyzed fillers is: 35%, 26%, 22%, and 19% for chokeberry pomace, raspberry seeds, hazelnut shells, and walnut shells, respectively, and increases with the increasing content of lignin in the filler.

#### 3.1.4. Analysis of the Filler Particles’ Surface Morphology

**Figure 3:**
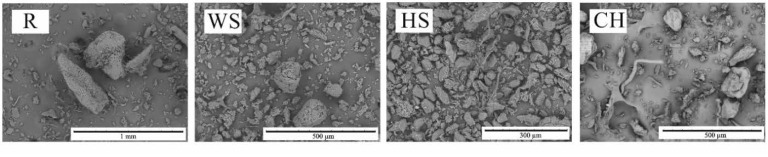
SEM images of the natural fillers.

Observations of the filler particles with a scanning electron microscope revealed that the fillers differed significantly in morphology (Figure 3). The ground raspberry seeds were characterized by a high content of particles with elongated irregular shapes and structures involving parallel fibers. Observations of the R grains showed their developed structure and porous surface. For this filler, a large proportion of fine particles resembling rugged flakes was also observed. The WS filler was also characterized by a high proportion of small irregular particles, but the shape of the observed large particles was quasispherical, with rough and smooth areas. A significant number of oval-shaped particles were observed in the hazelnut shells-based filler, as well as of helically twisted cellulose microfibrils. Large particles of chokeberry pomace were characterized by a lower degree of surface development compared to other fillers and by the presence of fibers and elongated fine particles.

#### 3.1.5. Analysis of Viscosity of the Polyols with the Natural Filler Particles

**Figure 4:**
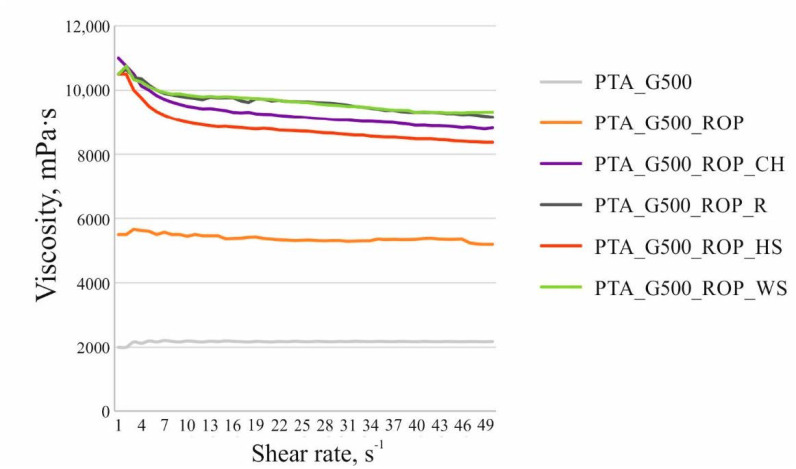
Viscosity curves for mixtures of polyols and mixtures of polyols with natural fillers. PTA_G500—the mixture of polyols Polios^®^ 420 PTA and Rokopol^®^ G500 in a 3:1 weight ratio; PTA_G500_ROP—the mixture of polyols Polios^®^ 420 PTA, Rokopol^®^ G500, and ROP in a 18.75:6.25:75 weight ratio (see Table 1); PTA_G500_ROP_CH/R/HS/WS—the mixture of polyols Polios^®^ 420 PTA, Rokopol^®^ G500, ROP, and natural fillers in a 18.75:6.25:75:15 weight ratio (see Table 1).

The replacement of 75 wt.% of petrochemical polyols with rapeseed polyol and the introduction of the natural filler particles into the PU composition resulted in an increase in viscosity of the systems (Figure 4). The highest increase in viscosity was observed after the incorporation of ground walnut shells and raspberry seeds. The reason for the difference in the increase in viscosity of the blends with HS and WS, despite the comparable particle size of both fillers, may be the higher proportion of irregular particles in the WS filler (Figure 3). The reason for the higher increase in viscosity when using R as compared to CH and HS may be the highest differentiation of the size of this filler and the high proportion of irregular rugged particles. The beginning of the shearing was accompanied by the decrease of dynamic viscosity of all of the modified systems. Once the filler molecules were arranged, the value of viscosity became relatively stable, which is a typical dependence for non-Newtonian fluids and has previously been observed for PU systems involving various kinds of organic and inorganic fillers [39].

#### 3.1.6. Analysis of the Foaming Process

The proper analysis of effects related to the introduction of natural components to the polyurethane composition requires a detailed investigation of the foaming process, which is the crucial part of the RPUF preparation. The size and shape of the cells formed during the foaming process have a critical impact on the mechanical and thermal insulating properties of foams [40].

**Figure 5:**
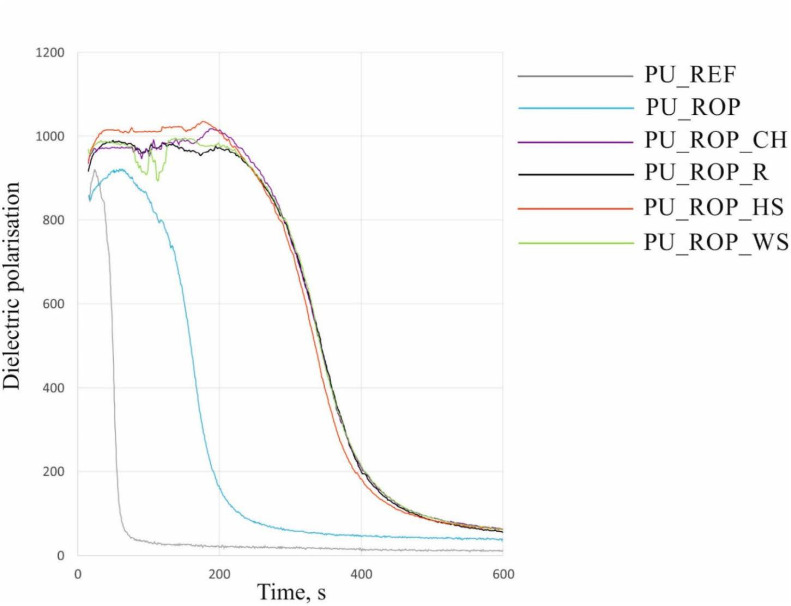
The influence of the ROP and natural fillers on the dielectric polarization during the foaming process.

**Figure 6:**
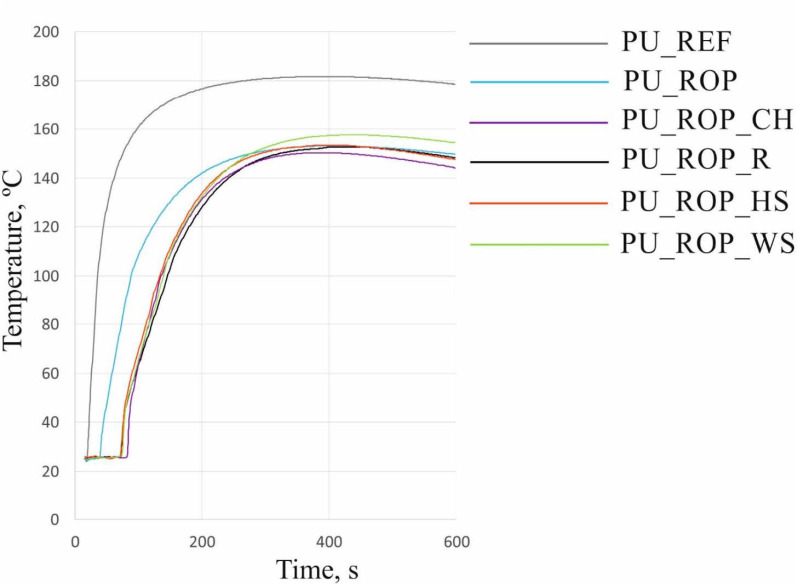
The influence of the ROP and natural fillers on the temperature during the foaming process.

One of the characteristic parameters describing the reactivity of a PU system is the dielectric polarization, which typically decreases as the reaction progresses [41]. Synthesis of a polyurethane foam conducted using only petrochemical substrates revealed the fastest decrease of the dielectric polarization value, which indicates the highest reactivity (Figure 5). The chemical transformation of this system was rapid, with an increase in the temperature of the foam core to 180 °C (which may lead to spontaneous combustion of the material in high-scale application) (Figure 6) [29]. The introduction of rapeseed polyol into the PU system resulted in reduced reactivity. These changes are related to the structure of the biopolyol, incorporating the secondary hydroxyl groups which are ca. three times less reactive than the primary hydroxyl groups [42,43]. The reduction in the reactivity of the system may also be caused by an increase in the viscosity of the reaction mixture after replacing petrochemical polyols with a high-viscosity vegetable polyol. The reactivity reduction of the PU system upon introduction of the biopolyol was also confirmed by the observation of decreased maximum temperature during the foaming process. Lowering the temperature inside the foam block to 155 °C for PU_ROP has a positive effect on the safety of manufacturing processes, as it reduces the risk of local overheating in the material and the risk of foam self-ignition. The higher viscosity of PU systems containing natural fillers hinders expansion of the reaction mixture and limits mobility of the molecules, which affects the reaction kinetics and slows down the rate of PU formation, which was also observed in the literature reports concerning the introduction of natural fillers such as nutmeg [4], oak bark [44], and wood fibers [45,46] into the PU mixture. The reason for the reduction in reactivity of the system could also be the slow release of water from the natural filler particles, hence the slower temperature rise in comparison to PU_REF and PU_ROP foams, where the total amount of water was introduced directly into the premix. The introduction of natural fillers caused slight changes in the maximum temperature in the block compared to the PU_ROP material (150 °C, 154 °C, 154 °C, and 158 °C for composites with CH, R, HS, and WS, respectively). This effect was different from the results presented in the work of Członka et al. where a significant increase in temperature was observed after the introduction of a natural filler in the form of potato protein. The temperature increase was explained by the presence of reactive groups in the filler (hydroxyl and amine) as well as the presence of residual water in the filler [23]. As a part of our study, in order to avoid excessive water in the reaction mixture, the amount of water that could be released during the reaction was initially determined and then included in the foam formulation.

### 3.2. Cell Structure and Properties of Rigid Polyurethane Foams

#### 3.2.1. Structure Analysis of the Polyurethane Composites

The structure of PU foams strictly depends on the formulation and adjustment of production parameters, including changes in viscosity and compatibility of raw materials. The cell structure and properties of polymeric composites are affected by the quality of the filler’s dispersion, its shape, size, surface development, and affinity to the polymer matrix. The addition of solid particles changes the nucleation process, influencing the number, size, and shape of the pores as well as the thickness of the walls and struts [25,30,36].

**Figure 7:**
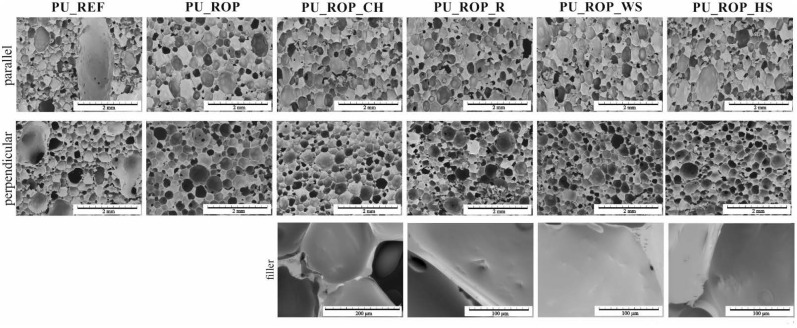
SEM images of the foams.

Changes in cell structures of the obtained materials were visualized by SEM images (Figure 7). More detailed photos demonstrating the filler particles in the composites are included in the supplementary file (Figure S5). The microscopic images of the PU_REF sample show a high proportion of irregularly shaped pores with a wide range of sizes (from 10 µm to 4000 µm), including cracks in the walls, which indicate open porosity, as well as a significant proportion of solid areas. Structural disturbances were probably the result of a rapid reaction with a significant increase in temperature in the foam block. The high amount of non-porous polyurethane as well as the disturbed structure of the material resulted in the highest apparent density (84 kg/m^3^, Table 1) of this material among the analyzed foams.

**Figure 8:**
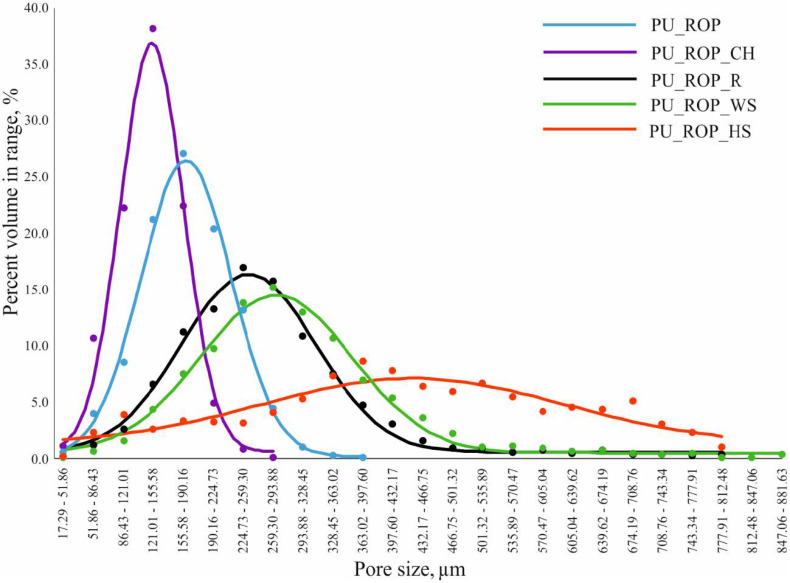
The pore size distribution for PU_ROP and PU_ROP_CH/R/WS/HS materials.

The replacement of 75 wt.% of petrochemical polyols with the rapeseed oil-based polyol resulted in the formation of a regular cell structure with smaller oval pores, which resulted from the lower reactivity of the system as well as lower temperature in the foam block compared to the PU_REF material. The formation of a more regular structure was also related to the fact that the rapeseed oil-based polyol acts is an additional surface-active agent which lowers the surface tension due to the presence of both hydrophobic (hydrocarbon fatty acids) and hydrophilic (ester groups, ether chains of DEG and hydroxyl groups) groups in the structure of the polyol. Obtaining a regular foam structure was associated with a decreased apparent density in comparison to the reference sample despite the use of the same amount of the blowing agent in the formulations. Moreover, the change of the apparent density of foams might also be related to the lower amount of isocyanate due to the lower hydroxyl value of the biopolyol [47]. The reduction of the pore size and the reduction of the apparent density after the incorporation of a plant polyol into the PU formulation were also reported in other scientific papers [30]. The introduction of the natural filler particles into the PU_ROP system resulted in the differentiation of the pore sizes. As demonstrated in previous reports, solid particles are likely to influence the rheology around the nucleating bubbles of gas, which reduces the nucleation energy, inducing the change of nucleation type from homogenous to heterogeneous. The low nucleation barrier facilitates the extensive formation of smaller pores, which later coalesce into larger units [4]. The SEM images indicate that the pore size of the foams produced with the use of the ROP and natural fillers was smaller compared to the pore size of the reference material. In order to acquire more detailed data concerning porosity, pore size, and filler size in foams, the RPUFs containing natural raw materials were tested using microtomography (CT). The analysis results indicate that the introduction of the chokeberry pomace into the foam formulation resulted in the decrease of the pore size (size range: 17–300 µm) in comparison to the PU_ROP material (size range: 17–400 µm), which indicates that the natural filler particles acted as nuclei for the growth of the new gas bubbles, leading to the formation of a structure containing a high number of fine pores (Figure 8), as evidenced by SEM observations (Figure 7).

**Figure 9:**
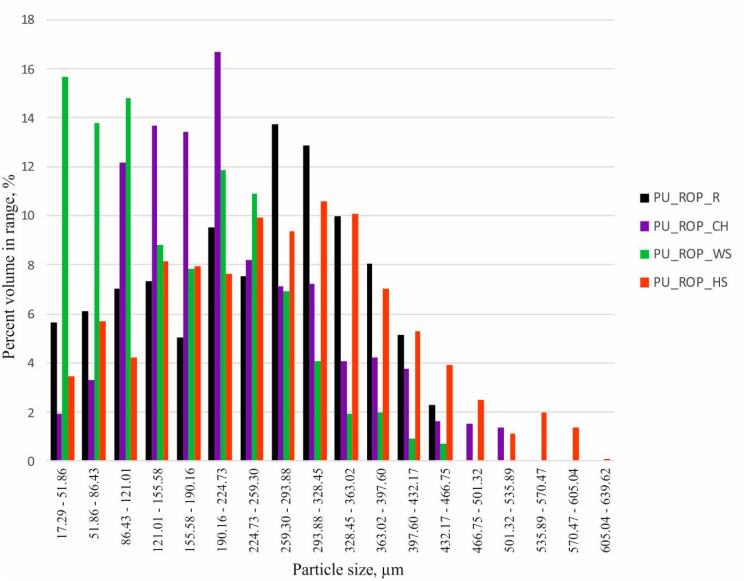
The filler size distribution for the PU_ROP_CH/R/WS/HS composites.

These results were found to be in agreement with the report by Silva et al. and Jonjaroen et al. where reduction of foam cells was demonstrated after the introduction of waste cellulose fibers and algal cellulose, respectively [22,46]. The use of the other fillers caused an increase in the size of the pores, especially in the case of hazelnut shells, followed by walnut shells and raspberry seeds. The increased cell size and pore size range in the case of PU_ROP_HS could be related to the distribution of the filler particles in the foam, which shows that ~50% of the HS particles exceeded 300 µm in size (Figure 9), while the sieve analysis report indicated 100% of the filler particles were smaller than 300 µm (Figure 1). It was described in previous literature reports that small particles tend to agglomerate leading to the formation of large clusters in the PU matrix [4]. The enhanced capabilities for gravitational migration of agglomerates could lead to the fusion of cell nuclei at the initial foaming stage, thus causing formation of large pores. Apart from the filler size, the tendency to form agglomerates is also influenced by its shape and surface development, as indicated by the results obtained for the foam with the WS filler where, despite the similar grain size of HS and WS fillers in the PU_ROP_WS material, a lower share of agglomerates was observed compared to the PU_ROP_HS material. Due to the higher degree of WS surface development compared to HS, the WS filler caused a greater increase in the viscosity of the polyol premix, which could lead to higher friction during the polyol premix mixing resulting in an improved dispersion of the filler. The foam containing the raspberry seeds was characterized by smaller cell diameters compared to the PU_ROP_HS and PU_ROP WS materials and by agglomeration of particles from the smallest fractions. The increase in the size of foam cells due to the incorporation of wood fibers was reported by Gu et al. [45]. The different influences of wood fibers in the publications of Gu et al. [45] and Silva et al. [46] could be related to the different morphology of the used fibers. In Silva’s report, the branched fiber residues were selected, while non-branched pulp fibers were used in the report by Gu et al. [45]. The introduction of natural fillers to the PU_ROP system led to an increase in the apparent density of materials, which resulted from the higher density of the fillers compared to the apparent density of foams [34]. A similar effect was reported in publications by Gu et al. [45], Silva et al. [46], and Jonjanoren et al. [22]. The PU_ROP_CH composite was characterized by the lowest apparent density among the foams with natural fillers which may be due to the fine cell structure of the material. The results of the computer microtomography also indicate an increase in the total porosity for materials with larger cell size (Table 3). The introduction of a natural polyol promoted the formation of closed cells in the produced materials as indicated by the closed cell analysis (Table 3). The introduction of the natural filler particles did not significantly affect the closed cell content in the prepared composites.

#### 3.2.2. Analysis of the Chemical Composition of the Foams

**Figure 10:**
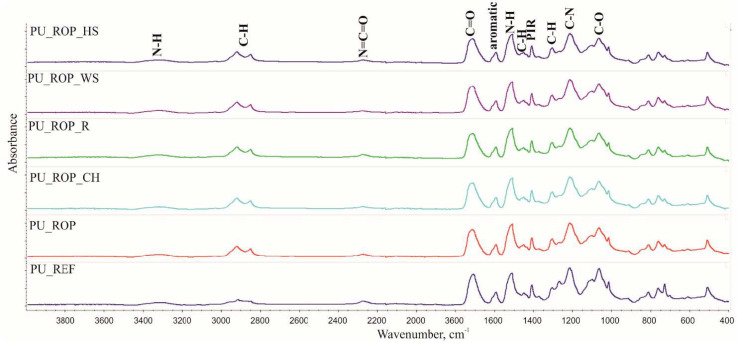
FTIR spectra of the foams.

The FTIR analysis was used in order to validate the presence of the characteristic groups in PU materials and to investigate the differences in the structure of the developed materials due to the introduction of the ROP and natural fillers into the PU composition. All of the expected characteristic signals originating from the polyurethane matrix were observed in the recorded FTIR spectra (Figure 10) [39,48]. The results of the FTIR study demonstrate the differences in the asymmetric and symmetric stretching vibrations of C–H bond regions in the reference material and the foams prepared using a natural polyol, which were related to the differences in the chemical structure of the polyols. No additional signals from the introduced natural fillers were observed because the bands from the plant-based fillers were present in the same range as the signals from the polyurethane matrix. Moreover, it was reported that the three main components of the biomass, cellulose, hemicellulose, and lignin, include alkenes, esters, aromatics, ketones, and alcohol moieties, as well as different oxygen-containing functional groups observed in the FTIR spectra, e.g., OH (3400–3200 cm^−1^), C=O (1765–1715 cm^−1^), C–O–C (1270 cm^−1^), and C–O–(H) (~1050 cm^−1^) [37].

#### 3.2.3. Thermal Analysis of the Rigid Polyurethane Foams

**Figure 11:**
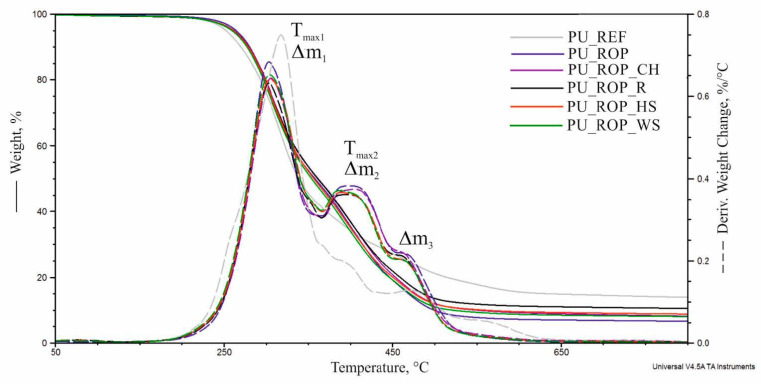
TG and DTG thermograms of foams.

**Figure 12:**
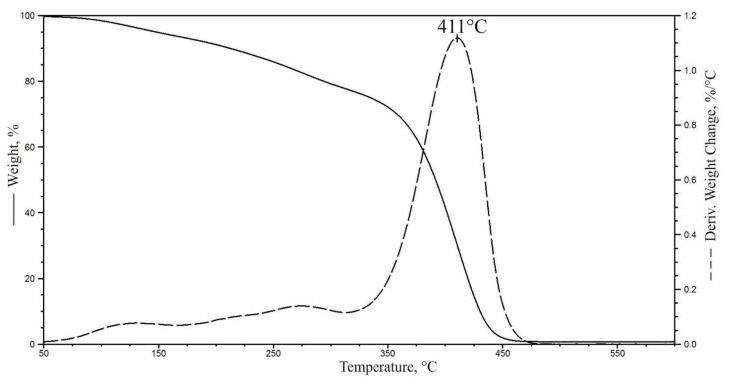
TG and DTG thermograms of the rapeseed oil-based polyol.

Based on the TG and DTG curves obtained in the thermogravimetric analysis, the degradation onset temperature (5% material loss, T_5%_) was determined for all of the samples as well as the amount of residue after combustion at 800 °C (R), the temperature of the maximum degradation rate (T_max_) along with the degradation rates (V_max_) and weight loss in the subsequent stages of thermal decomposition of foams (Δm) (Table 4, Figure 11). The results show that the use of a plant-based polyol for the synthesis of PU foams caused an increase in the thermal degradation onset temperature by 12 °C, which may result from the higher thermal stability of the rapeseed polyol (Figure 12) compared to petrochemical polyols [6]. The introduction of natural fillers to the PU system resulted in a slight decrease in T_5%_ (3–5 °C), but the thermal degradation onset temperature of the composites remained higher compared to PU_REF.

Analysis of the DTG curve of the reference material shows one major foam degradation stage in the temperature range 200–450 °C with a maximum decomposition rate of 0.75%/°C at 317 °C. This stage is related to the thermal degradation of the rigid and flexible segments of the material [9]. In the temperature range of 450–650 °C, thermolysis of organic residues occurs [6], accompanied by the weight loss of Δm_3_ = 12%. Partial replacement of petrochemical polyols with the rapeseed polyol resulted in changes in the structure of foams and, consequently, in the course of the DTG curve. As a result, the signal related to the degradation of flexible segments made of rapeseed polyol (T_max2_) was isolated in the temperature range of 366–450 °C, with a weight loss Δm_2_ = 28%, which consequently reduced the values of Δm_1_ and V_max1_. The residue after combustion (R_800_) decreased after the introduction of the ROP, as reported in the previous study in the field [6]. The introduction of plant-based fillers to the PU_ROP system caused changes in the course of the DTG curve in the temperature range of 366–450 °C due to the differences in the chemical composition of the fillers. As the proportion of lignin in a filler increases, the maximum of the T_max2_ signal shifts towards higher temperatures. The residue after burning the composites was higher compared to the PU_ROP material.

**Figure 13:**
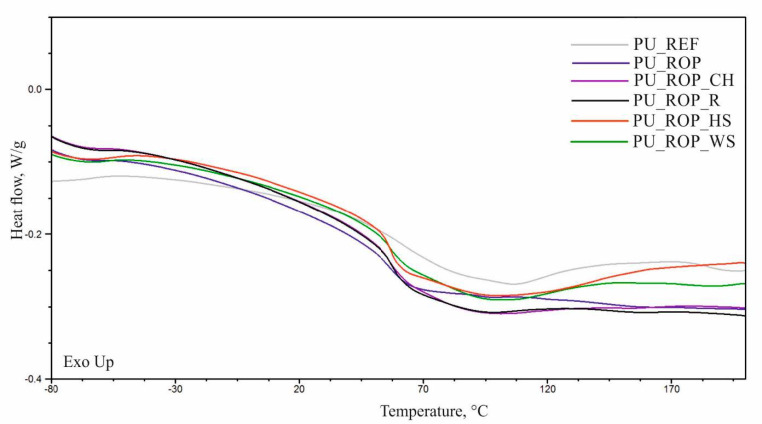
DSC curves (C1) of the developed materials.

**Figure 14:**
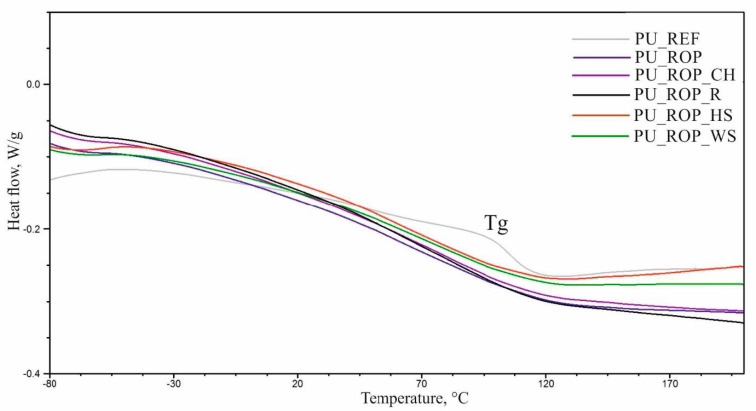
DSC curves (C2) of the developed materials.

The temperatures of the physical phase transitions were determined using the DSC analysis. The DSC curves of the first heating cycle (C1) revealed a presence of an endothermal transition peak in the range of 40–140 °C corresponding to the order–disorder transformation in polyurethane foams (Figure 13). The glass transition temperature of the hard phase (Tg) was determined using the data of the second heating cycle (C2) (Table 5, Figure 14). The thermogram of the reference material showed a more rapid change in heat flow in the glass transition temperature region in comparison to the thermograms of foams produced with the use of the ROP. The increased content of the ROP resulted in a significant decrease of the Tg from 105 °C (PU_REF) to 84 °C (PU_ROP) as a result of the plasticizing effect of the hydrocarbon chains present in the unsaturated fatty acids from the rapeseed oil-based polyol [12]. The introduction of natural fillers reduces mobility of the rigid segments, which is indicated by the increase in the glass transition temperature of the composites. These results indicate that during the formation of foams, the NCO groups react with the functional groups of the filler components, stiffening the rigid segments. The increase in the glass transition temperature after the introduction of a vegetable filler was reported in the work by Gu et al. [45].

#### 3.2.4. Analysis of the Physicomechanical Properties of the Foams

**Figure 15:**
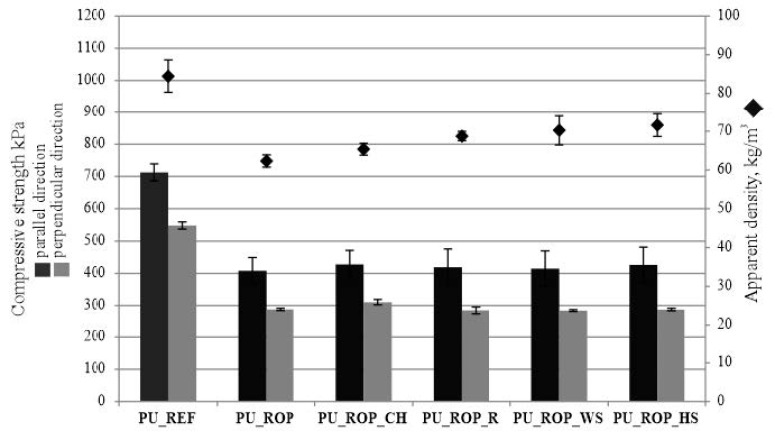
The compressive strength and apparent density of the RPUFs.

The introduction of the ROP in the polyol premix led to a decrease in compressive strength due to the plasticizing influence of the rapeseed oil-based polyol as well as a decrease in the apparent density of resulting composites (Figure 15) [29]. The reason for the high compressive strength of PU_REF may also be related to solid areas in the material and its higher apparent density. The compressive strength of composites with natural fillers is comparable to the compressive strength of the PU_ROP material; however, the foams containing natural fillers were characterized by a higher apparent density compared to the neat foam based on the ROP; therefore, in order to better present the relationship between compressive strength and apparent density of fillers, specific compressive strength (ratio of compressive strength to apparent density) was determined (Supplementary Data, Table S5). The results of the specific compressive strength analysis indicate that the introduction of R, WS, and HS fillers decreased this value. The observed decrease in the specific compressive strength was associated with the presence of defects in the materials resulting from the change in the morphology and the presence of more irregular cells compared to the PU_ROP material. The reduction in compressive strength after the incorporation of cellulose fibers (3–16 php) was reported in the publication by Silva et al. [46]. A similar effect was obtained in the article by Gu et al. [45] after the introduction of wood fibers (13.1 php) and in the work of Paciorek–Sadowska et al. [44], where the authors used oak bark for the production of foams, justifying the reduction of compressive strength by the presence of irregular pores and structure disturbances. A higher value of specific compressive strength of the PU_ROP_CH material resulted from the regular foam structure and the high share of small pores. The introduction of the rapeseed oil-based polyol resulted in the lower friability of the foam compared to the reference sample, which was likely a result of the plasticizing effect of the vegetable polyol (Table 6). On the other hand, the introduction of natural fillers caused its slight increase in comparison to the PU_ROP material, which might be related to the chipping of the natural filler particles together with the PU matrix. All of the materials produced using the renewable raw materials exhibited lower friability compared to the material produced using only petrochemical raw materials. The dimensional stability of the materials containing the ROP increased. The water absorption decreased due to the hydrophobic characteristics of fatty acids within the natural oil-based polyols. The bio-materials produced within the study are characterized by low water absorption <1% and high dimensional stability < ±0.5%. Moreover, as evidenced in the study, the chemical composition of the natural fillers had no effect on the dimensional stability and water absorption, which resulted from the total immersion of the filler particles in the polymer matrix. Similar observations were described in the work of Silva et al., where it was indicated that the cellulose fibers used for the production of rigid polyurethane foam composites were dispersed in the interior and in the borders of the cells recovered by the polymer, which resulted in the absence of moisture uptake by the foams determined on the basis of thermogravimetric analysis [46].

## 4. Conclusions

The paper demonstrates the development of rigid polyurethane foam composites using 75 wt.% of the rapeseed oil-based polyol and 15 php of natural fillers such as chokeberry pomace, raspberry seeds, as well as hazelnut and walnut shells. Introduction of renewable substrates resulted in the decrease in the foaming mixture reactivity, which led to a lower maximum temperature of the foaming process from 180 °C observed for the reference material prepared using 100% petrochemical substrates to ~155 °C for polyurethane biofoams. Moreover, the introduction of natural compounds resulted in the formation of materials with a more regular cell structure and a higher share of closed cells. Significant differences in the structure of the composites were observed depending on the type of filler used, which was related to the differences in the grain size, its shape, and the degree of surface development. The introduction of the ROP to the polyol premix led to a decrease in compressive strength of the materials with regard to the plasticizing effect of the rapeseed oil-based polyol as well as a decrease in the apparent density of modified foams. The compressive strength of the composites with natural fillers was comparable to the compressive strength of the material with the vegetable polyol. The materials produced with the use of renewable raw materials were characterized by low friability < 2%, low water absorption <1%, and high dimensional stability < ±0.5%. Water absorption decreased and the dimensional stability of the foams increased upon the replacement of 75 wt.% petrochemical polyols with the rapeseed polyol. The introduction of natural components resulted in increased thermal stability of the developed foams. The introduction of plant-based fillers to polyurethane systems resulted in differences in the course of thermal degradation of foams due to differences in the chemical composition of the fillers. The use of the rapeseed polyol reduced the glass transition temperature of the foams. In turn, the introduction of natural fillers led to an increase in the glass transition temperature due to a reduction in the mobility of the rigid segments in the material. Overall, the results obtained in the study indicate that the proper selection and preparation of the natural raw materials as well as the rational development of the RPUF composition and synthesis method allow for the preparation of environmentally friendly materials with valuable properties in compliance with the postulates of the circular economy in the synthesis of polyurethane foams.

## Figures and Tables

**Table 1:** Summary of RPUF formulations used in the study (amounts in parts by weight - pbw).

Sample	PU_REF	PU_ROP	PU_ROP_CH	PU_ROP_R	PU_ROP_WS	PU_ROP_HS
Raw Materials	Composition (pbw)					
Natural filler	-	-	15	15	15	15
Polios^®^ 420 PTA	75	18.75	18.75	18.75	18.75	18.75
Rokopol^®^ G500	25	6.25	6.25	6.25	6.25	6.25
ROP	0	75	75	75	75	75
Jeffcat^®^ DPA	1	1	1	1	1	1
Jeffcat^®^ ZF-10	0.55	0.55	0.55	0.55	0.55	0.55
Distilled water	1.35	1.30	1.01	1.03	1.00	1.00
Tegostab^®^ B4900	1.25	1.25	1.25	1.25	1.25	1.25
Ongronat^®^ TR 4040	Isocyanate index 110					
Apparent density kg/m^3^	84 ± 4	62 ± 2	65 ± 2	69 ± 1	70 ± 4	72 ± 3

**Table 2:** The chemical composition of the natural fillers.

Natural Filler	Hemicellulose, %	Cellulose, %	Lignin, %	Raw fat, %
CH	21.7	20.6	58.0	7.3
R	38.5	28.0	42.3	12.2
HS	20.3	37.8	38.7	1.5
WS	28.6	34.7	37.3	5.5

**Table 3:** Closed cell content (ISO 4590) and total porosity (CT) of the analyzed materials.

Sample	Closed Cell Content, %	Total Porosity, %
PU_REF	84 ± 1	-
PU_ROP	89 ± 0	92
PU_ROP_CH	91 ± 0	89
PU_ROP_R	90 ± 0	95
PU_ROP_WS	89 ± 1	97
PU_ROP_HS	89 ± 1	94

**Table 4:** The results of the thermogravimetric analysis.

Sample	T_5%_, °C	T_max1_/_(_V_max1)_, °C/%/°C	Δm_1_, %	T_max2/_ V_max2_, °C/%/ °C	Δm_2_, %	Δm_3_, %	R (800 °C), %
PU_REF	252	317/0.75	72	-	-	12	14
PU_ROP	264	303/0.68	52	399/0.38	28	12	7
PU_ROP_CH	261	306/0.64	50	403/0.37	28	12	8
PU_ROP_R	261	303/0.63	52	397/0.36	27	11	11
PU_ROP_WS	259	304/0.65	53	386/0.37	27	11	8
PU_ROP_HS	261	305/0.64	52	388/0.37	27	11	9

**Table 5:** The results of the DSC curve analysis of the examined materials.

Sample	Tg, °C
PU_REF	105 ± 0
PU_ROP	84 ± 1
PU_ROP_CH	94 ± 1
PU_ROP_R	95 ± 2
PU_ROP_WS	90 ± 1
PU_ROP_HS	87 ± 2

**Table 6:** Friability, dimensional stability and water absorption of the examined materials.

Material	Friability, %	Dimensional Stability in Water, Thickness (24 h, 40 °C), %	Dimensional Stability in Water, Width (24 h, 40 °C), %	Dimensional Stability in Water, Length (24 h, 40 °C), %	Water Absorption (24 h, 40 °C), %
PU_REF	2.09 ± 0.8	−0.93 ± 0.02	−0.35 ± 0.49	−0.59 ± 0.23	2.68 ± 1.65
PU_ROP	0.31 ± 0.1	−0.07 ± 0.01	0.39 ± 0.33	0.47 ± 0.27	0.95 ± 0.14
PU_ROP_CH	1.41 ± 0.2	−0.22 ± 0.05	−0.21 ± 0.95	−0.25 ± 0.58	0.79 ± 0.18
PU_ROP_R	1.61± 0.2	0.04 ± 0.16	−0.28 ± 0.08	0.06 ± 0.14	0.74 ± 0.13
PU_ROP_WS	1.79 ± 0.3	−0.07 ± 0.06	−0.45 ± 0.49	0.42 ± 0.43	0.97 ± 0.09
PU_ROP_HS	1.94 ± 0.4	−0.12 ± 0.08	−0.28 ± 0.51	0.49 ± 0.75	0.91 ± 0.08

## Data Availability

The data presented in this study are available on request from the corresponding author.

## Supplementary Materials

The following are available online at https://www.mdpi.com/article/10.3390/ma14071772/s1, Figure S1: SEM images of the rigid polyurethane foams with rapeseed oil-based polyol, Figure S2: Results of the closed cell content analysis in PU_REF and PU_ROP_25-100 foams, Figure S3: TG and DTG thermograms of the rigid polyurethane foams with rapeseed oil-based polyol, Figure S4: DSC curves (C2) of the rigid polyurethane foams with rapeseed oil-based polyol, Figure S5: SEM images filler particles in the foams with rapeseed oil-based polyol and natural fillers, Table S1: The composition of rigid polyurethane foams, Table S2: The results of the thermogravimetric analysis, Table S3: The results of the DSC curve analysis of the rigid polyurethane foams with rapeseed oil-based polyol, Table S4: Apparent density, friability, dimensional stability and water absorption of the foams with rapeseed oil-based polyol, Table S5: Specific compressive strength of the foams with rapeseed oil-based polyol and natural fillers.
