# Supplementary material for: Vegetable Fillers and Rapeseed Oil-Based Polyol as Natural Raw Materials for the Production of Rigid Polyurethane Foams
Source: Materials (Basel). 2021 Apr 3;14(7):1772. doi: 10.3390/ma14071772 (PMC8038401; doi:10.3390/ma14071772)
Supplement: Supplementary file 1 [file materials-14-01772-s001.pdf]

materials

MDPI

Supplementary Materials: Vegetable Fillers and Rapeseed Oil-Based Polyol as Natural Raw Materials for the Production of Rigid Polyurethane Foams

Milena Leszczyńska, Elżbieta Malewska, Joanna Ryszkowska, Maria Kurańska, Michał Gloc, Michał K. Leszczyński and Aleksander Prociak

Section S1. Selection of the Optimal Content of Rapeseed Oil-based Polyol for Modification with Vegetable Fillers

Preparation of the Rigid Polyurethane Foams with Rapeseed Oil-based Polyol

The synthesis of the RPUFs was performed using a single-step method described in the manuscript paragraph 2.2 according to the recipes in Table S1.

Table S1. The composition of rigid polyurethane foams.

Sample	PU_REF	PU_ROP_25	PU_ROP_50	PU_ROP_75	PU_ROP_100
**Raw materials**			**Composition (pbw)**		
Polios®420PTA	75	56.25	37.5	18.75	0
Rokopol®G500	25	18.75	12.5	6.25	0
Rapeseed polyol	0	25	50	75	100
Jeffcat® DPA	1	1	1	1	1
Jeffcat® ZF-10	0.55	0.55	0.55	0.55	0.55
Distilled water	1.35	1.33	1.32	1.30	1.29
Tegostab® B4900	1.25	1.25	1.25	1.25	1.25
Ongronat® TR 4040			Isocyanate index 110		

Analysis of the Structure of the Rigid Polyurethane Foams with Rapeseed Oil-based Polyol

The replacement of 25–75 wt.% of petrochemical polyols with rapeseed oil-based polyol resulted in the formation of a regular cell structure with smaller oval pores (Figure S1). The complete replacement of petrochemical polyols with ROP caused the widening of the cell size range, which may be due to the high viscosity of the polyurethane system.

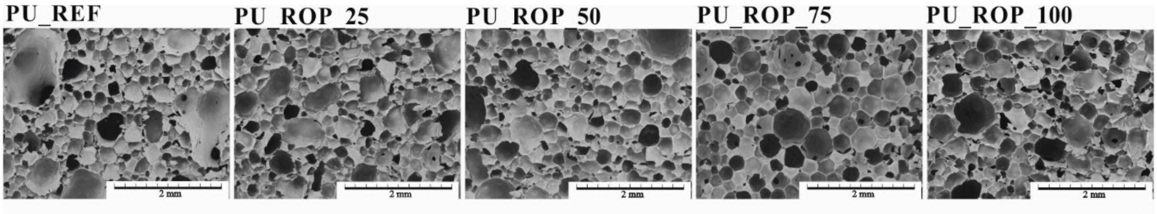

Figure S1. SEM images of the rigid polyurethane foams with rapeseed oil-based polyol.

Analysis of the Closed-Cell Content in the Rigid Polyurethane Foams with Rapeseed Oil-based Polyol

The increasing content of ROP (25–75 wt.%) in the polyol premix resulted in the increased closed cell content in the foams (Figure S2). The complete replacement of petrochemical polyols with vegetable polyol resulted in a decrease in the closed cell content.

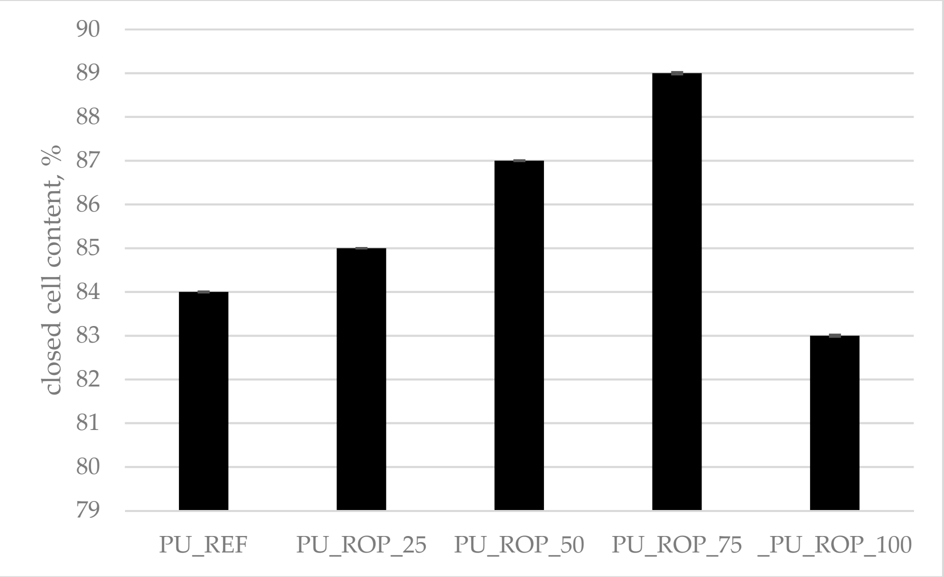

Figure S2. Results of the closed cell content analysis in PU_REF and PU_ROP_25-100 foams.

Thermal Analysis of the Rigid Polyurethane Foams with Rapeseed Oil-based Polyol

The results of the thermogravimetric analysis show that the temperature of the onset of thermal degradation ($T_{5\%}$) increases with the increased content of ROP in the foams (Figure S3, Table S2). The residue after combustion at 800 °C decreases along with the increase in the proportion of ROP in the foams.

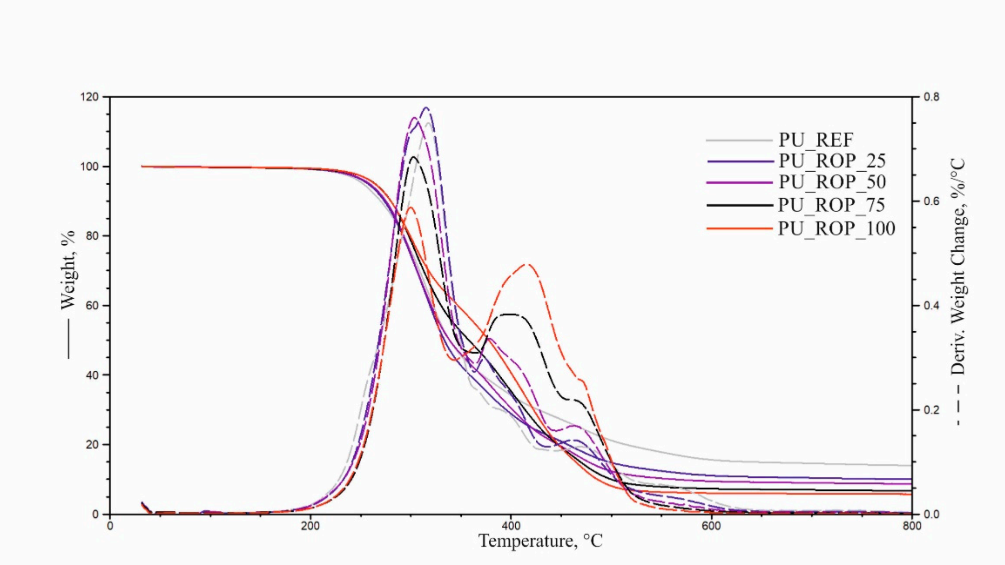

Figure S3. TG and DTG thermograms of the rigid polyurethane foams with rapeseed oil-based polyol.

Table S2. The results of the thermogravimetric analysis.

Sample	$T_{5\%}$, °C	T_{max1}, °C	T_{max2}, °C	R (800°C),%
PU_REF	252	317	-	14
PU_ROP_25	257	315	373	10
PU_ROP_50	257	308	378	8
PU_ROP_75	264	303	397	7

PU_ROP_100	264	300	415	6

The results of the analysis performed with the use of differential scanning calorimetry indicate the glass transition temperature of the hard phase (Tg) decreases with the increased content of ROP in the foams (Figure S4, Table S3). The reason for the plasticizing effect is the participation of unsaturated fatty acid hydrocarbon chains in the ROP structure.

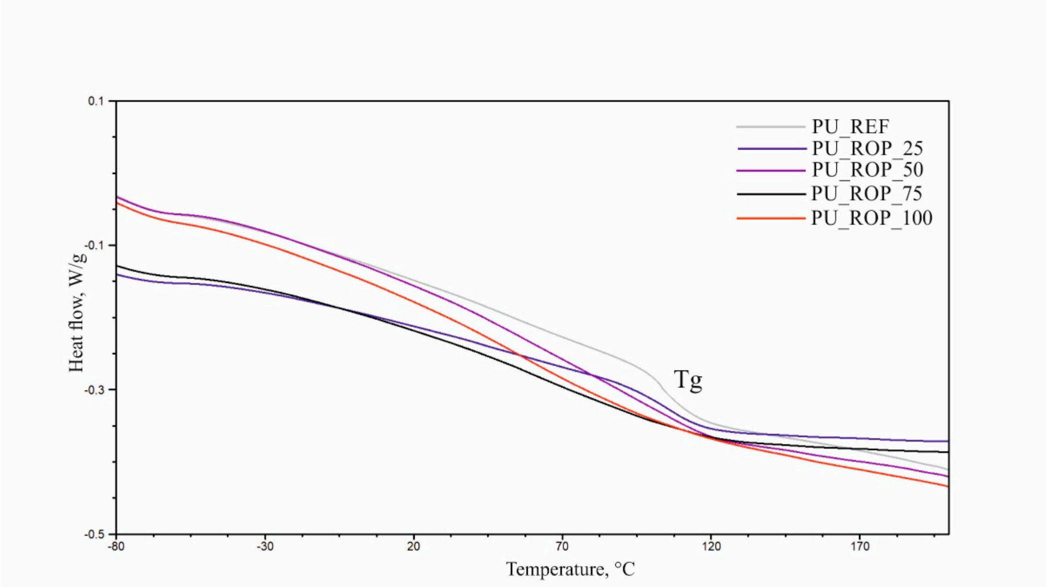

Figure S4. DSC curves (C2) of the rigid polyurethane foams with rapeseed oil-based polyol.

Table S3. The results of the DSC curve analysis of the rigid polyurethane foams with rapeseed oil-based polyol.

Sample	Tg, °C
PU_REF	105
PU_ROP_25	105
PU_ROP_50	91
PU_ROP_75	84
PU_ROP_100	84

Analysis of the Physico-Mechanical Properties of the Foams with Rapeseed Oil-based Polyol

Table S4. Apparent density, friability, dimensional stability and water absorption of the foams with rapeseed oil-based polyol.

Sample	Apparent Density, kg/m³	Friability, %	Dimensional Stability in Water, Thickness (24 h, 40°C), %	Dimensional Stability in Water, Width (24 h, 40°C), %	Dimensional Stability in Water, Length (24 h, 40°C), %	Water Absorption (24 h, 40 °C). %
PU_REF	84 ± 4	2.1 ± 0.8	−0.93 ± 0.02	−0.35 ± 0.49	−0.59 ± 0.23	2.68 ± 1.65
PU_ROP_25	69 ± 4	0.6 ± 0.3	−0.66 ± 0.09	0.29 ± 0.14	−0.29 ± 0.39	0.90 ± 0.26

PU_ROP_50	68 ± 2	0.4 ± 0.2	−0.56 ± 0.10	0.72 ± 0.32	0.69 ± 0.11	0.69 ± 0.17
PU_ROP_75	62 ± 2	0.3 ± 0.1	−0.07 ± 0.01	0.39 ± 0.33	0.47 ± 0.27	0.95 ± 0.14
PU_ROP_100	57 ± 1	1.5 ± 0.3	−0.27 ± 0.17	0.49 ± 0.29	0.85 ± 0.52	1.06 ± 0.23

The results of the analysis indicate that increasing the content of ROP in the material causes a decrease in apparent density (Table S4). The use of rapeseed oil-based polyol also reduced the friability of the materials (the lowest for PU_ROP_75 material) and reduced water absorption. The produced materials were characterized by high dimensional stability (< ±1%).

Summary of the Results of the Analysis the Rigid Polyurethane Foams with Different Ratios of Rapeseed Oil-based Polyol

Due to the high content of renewable raw material in the foam as well as regular structure, high content of closed cells, low apparent density, low friability, low water absorption, high dimensional and thermal stability, a foam prepared using 75 wt.% rapeseed oil-based polyol (PU_ROP_75) was selected for modification with vegetable fillers.

Section S2.Supplementary Results for Foams with Rapeseed Oil-based Polyol and Natural Fillers

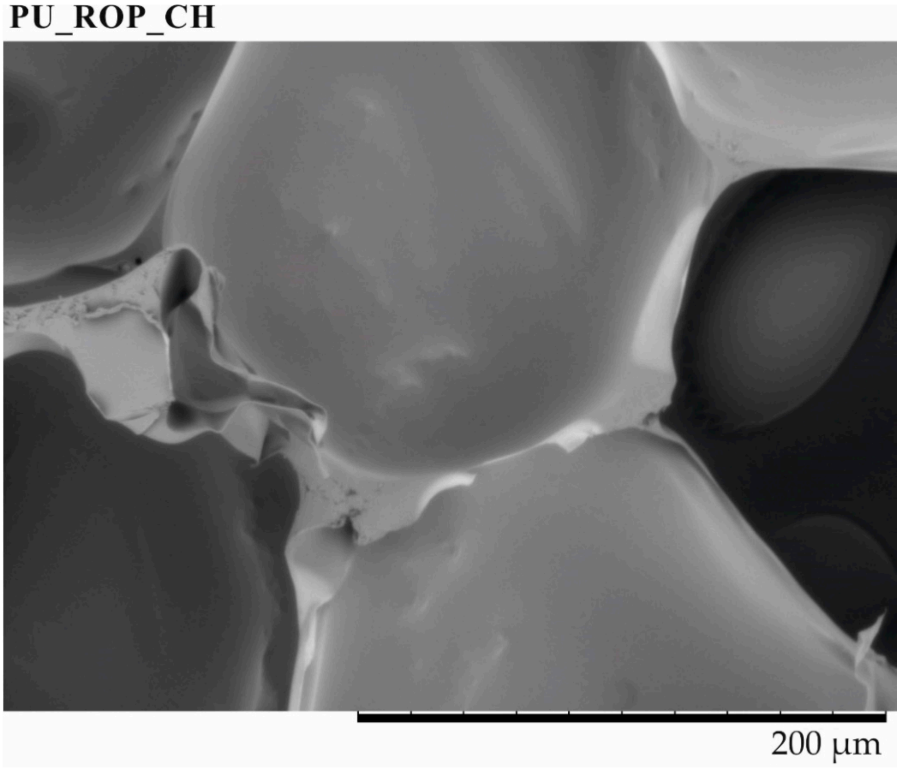

materials

PU_ROP_R

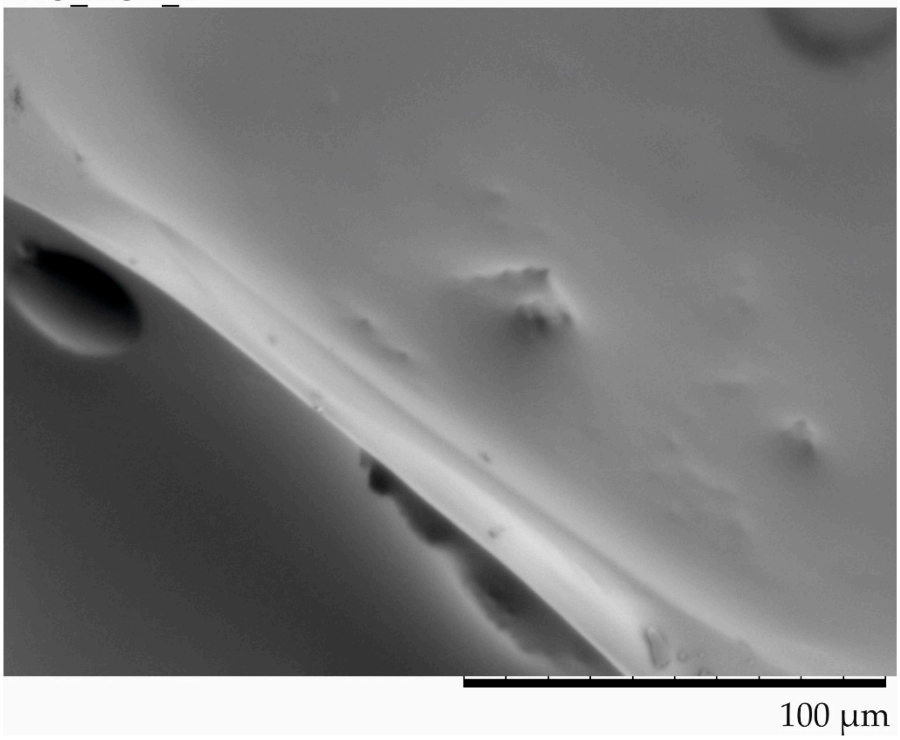

PU_ROP_WS

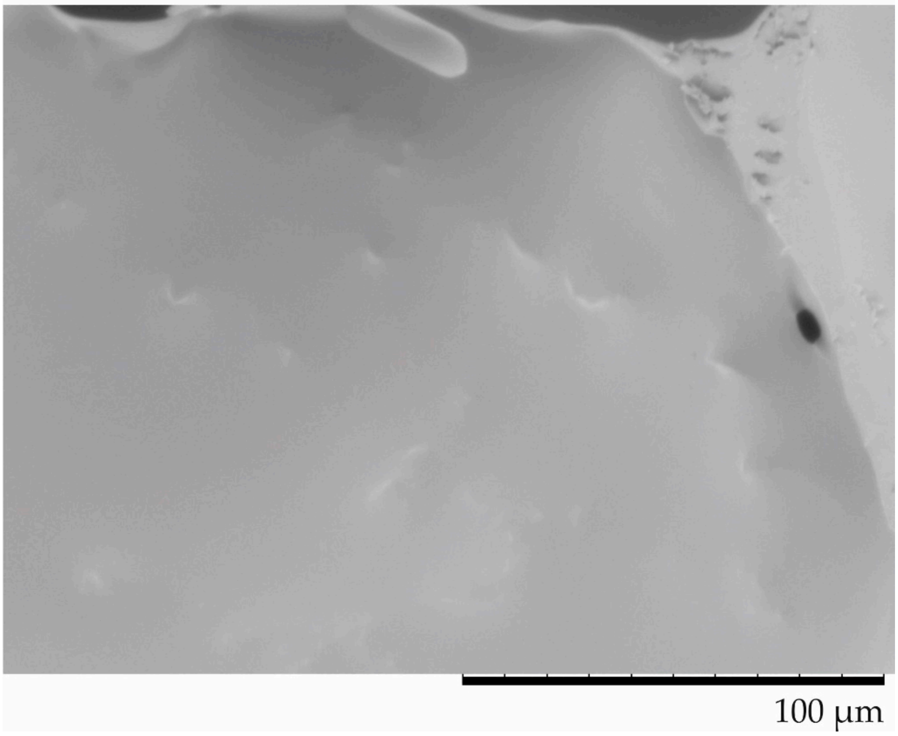

PU_ROP_HS

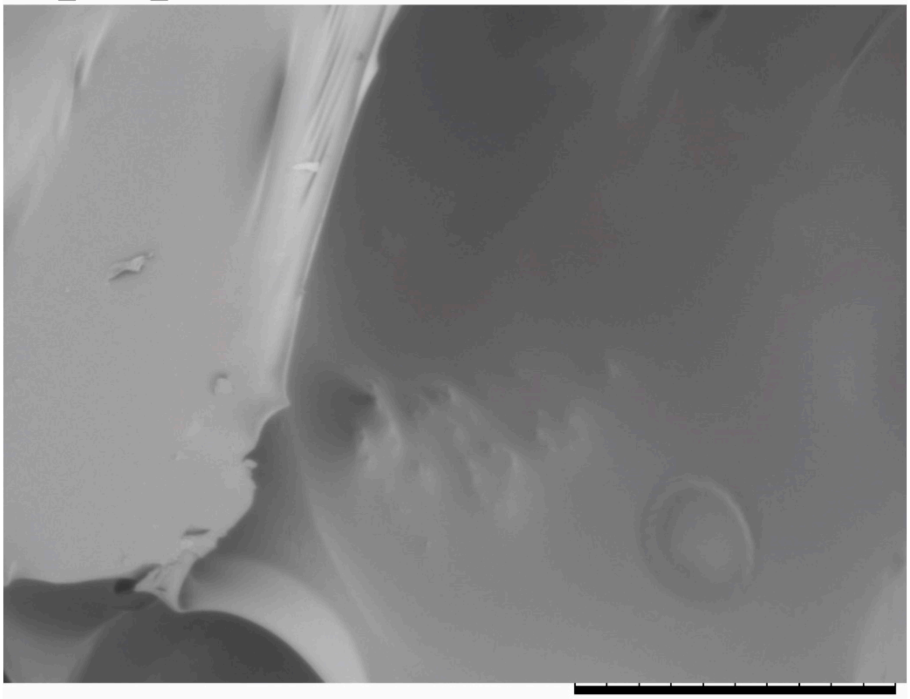

Figure S5. SEM images filler particles in the foams with rapeseed oil-based polyol and natural fillers.

Table S5. Specific compressive strength of the foams with rapeseed oil-based polyol and natural fillers.

Sample	Specific Compressive Strength – Parallel, kPa m³/kg	Specific Compressive Strength – Perpendicular, kPa m³/kg
PU_REF	8.44	6.49
PU_ROP	6.55	4.63
PU_ROP_CH	6.54	4.75
PU_ROP_R	6.10	4.15
PU_ROP_WS	5.91	4.06
PU_ROP_HS	5.96	4.02
